# The Role of the Immune Response in the Pathogenesis of Bronchiectasis

**DOI:** 10.1155/2018/6802637

**Published:** 2018-03-18

**Authors:** Paul T. King

**Affiliations:** Monash Lung and Sleep and Monash University Department of Medicine, Monash Medical Centre, 246 Clayton Rd, Clayton, Melbourne, VIC 3168, Australia

## Abstract

Bronchiectasis is a prevalent respiratory condition characterised by permanent and abnormal dilation of the lung airways (bronchi). There are a large variety of causative factors that have been identified for bronchiectasis; all of these compromise the function of the immune response to fight infection. A triggering factor may lead to the establishment of chronic infection in the lower respiratory tract. The bacteria responsible for the lower respiratory tract infection are usually found as commensals in the upper respiratory tract microbiome. The consequent inflammatory response to infection is largely responsible for the pathology of this condition. Both innate and adaptive immune responses are activated. The literature has highlighted the central role of neutrophils in the pathogenesis of bronchiectasis. Proteases produced in the lung by the inflammatory response damage the airways and lead to the pathological dilation that is the pathognomonic feature of bronchiectasis. The small airways demonstrate infiltration with lymphoid follicles that may contribute to localised small airway obstruction. Despite aggressive treatment, most patients will have persistent disease. Manipulating the immune response in bronchiectasis may potentially have therapeutic potential.

## 1. Introduction

Bronchiectasis is characterised by permanent and abnormal dilation of the lung airways (bronchi). It arises from persistent bacterial airway infection on a background of a deficient immune response. The consequent inflammatory response to infection is largely responsible for the pathology of this condition.

Bronchiectasis is a condition that for many years has had a low profile and had been designated as being an “orphan disease” [1]. However, the widespread availability of high resolution computed tomography (HRCT) scanning has led to a realisation that it is a common condition and a leading cause of respiratory morbidity and mortality. The prevalence of bronchiectasis is not clearly defined. Weycker et al. reported that between 340,000 and 522,000 adults in the US population were receiving treatment for bronchiectasis and that 70,000 adults were newly diagnosed with bronchiectasis in 2013 [2]. Another study reported that there were more than two million adults with bronchiectasis worldwide in 2012 and this was expected to increase to more than three million by 2020 [3]. In addition, it has been recently been recognised that bronchiectasis frequently occurs in patients with chronic obstructive pulmonary disease (COPD). Up to 50% of patients with COPD may have coexistent bronchiectasis [4–6].

From the immunology point of view, bronchiectasis is of significant interest as it provides insights into both mechanisms of immune deficiency and the consequent persistent inflammatory response to bacterial infection. It also provides a potential opportunity to manipulate the immune response to improve patient outcome. It should be emphasised that there are a wide variety of factors that may contribute to the development of bronchiectasis and the pathogenesis is still not fully understood (Table 1).

Cystic fibrosis (CF) is characterised by severe bronchiectasis but this review will only deal with non-CF bronchiectasis.

## 2. Pathology

Patients with bronchiectasis have their airways colonised by bacteria which activates the immune response with consequent inflammation. Arguably the most important study of the pathogenesis was performed by Whitwell in the 1950s [7]. He obtained 200 lobectomy samples from patients. The main site of inflammation was in the small airways (terminal bronchi/bronchioles) which had a dense infiltrate of mononuclear cells with the formation of lymphoid follicles. Bronchiectasis occurred in the larger airways with destruction of the connective tissue/cartilage (arising from protease effects on the lung tissue), which resulted in the large airway dilation. In contrast in the smaller airways, the cell wall infiltrate resulted in bronchial narrowing and obstruction.

**Figure 1:**
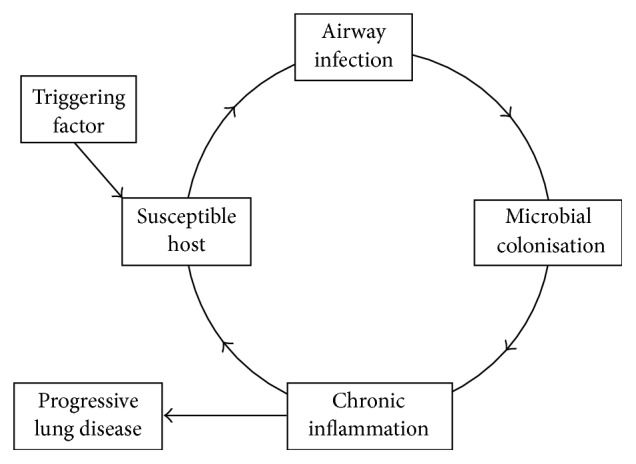
*The vicious circle of bronchiectasis.* As modified from Cole's work [8].

Cole proposed the “vicious circle” of bronchiectasis [8] (also has been termed cycle). In this model, a triggering event occurs on a background of host susceptibility (i.e., some form of defective host defence). This results in persistent infection in the lower respiratory tract causing chronic inflammation and progressive lung damage. A key feature of this model is that once bacterial infection/inflammation is established in the lung, it will persist indefinitely. In his initial description, Cole emphasised the central role of bacterial exotoxins and ciliary dysfunction. The current interpretation does not emphasise this factor as much and a representation is shown in Figure 1. Clinically most patients with bronchiectasis tend to have persistent symptoms and have a gradual decline in their lung function [9–12]. The triggering factor in this model has not been well defined. Another factor identified by Cole in the pathogenesis of bronchiectasis was obstruction, particularly in the context of being unable to clear sputum adequately which led to more infection and subsequent inflammation [9].

Similar to COPD, bronchiectasis is also characterised by acute exacerbations in which there are acute episodes of increased symptoms often requiring a change in medication [13, 14]. Most commonly, these episodes are driven by increased airway inflammation. Acute exacerbations of COPD are a major driver of morbidity and mortality and contribute to lung function decline. It is likely that acute exacerbations have a similar effect in bronchiectasis, although this has not been definitively proven.

## 3. Microbiology

Most of the bacteria that cause pulmonary inflammation in bronchiectasis are present as commensals in the microbiome of the upper respiratory tract, concentrated in the nasopharynx. The respiratory microbiome becomes established in the first year of life and this is a dynamic process with turnover of different species and strains [15]. Some of the bacteria in the microbiome may cause inflammation when they move into the lower respiratory tract and may be designated as the potential pathogenic microorganisms (PPMs) [16]. The reasons for this proinflammatory effect in the lung for these PPMs are not well understood. Important bacteria in the context of bronchiectasis are* Haemophilus influenzae* (generally the nontypeable form (NTHi)),* Streptococcus pneumoniae, Moraxella catarrhalis, Mycobacterial *spp., and* Pseudomonas aeruginosa*. Infection with* P. aeruginosa* usually occurs in more advanced disease. These bacteria use a variety of strategies to prevent clearance by the lung immune defence, which include inhibiting mucociliary clearance and the formation of biofilms [17, 18].

The most extensive microbiome is present in the gastrointestinal tract (GIT). Trafficking of immune cells from the GIT to other sites in the body occurs and may influence local immune responses. Changes in diet and GIT microbiota influence allergic airways disease [19].

## 4. Causes of Bronchiectasis

Deficiency of the host immune response to bacterial infection is regarded as a primary requirement for the development of bronchiectasis. There have been a large number of causes for bronchiectasis identified. All of these etiologic factors compromise the host defence to infection in some way. In addition, many patients do not have an identifiable cause and are designated as having idiopathic disease [20, 21]. Most patients have symptoms for many years and the condition often arises in early childhood; therefore the absolute role of identified etiologic factors is often not clear. They could perhaps be considered to be risk factors, in a similar way to other chronic diseases which are multifactorial (e.g., ischemic heart disease).

### 4.1. Postinfective

The most commonly described cause for the development of bronchiectasis is postinfective. Childhood infections such as whooping cough and measles have been identified as important causes. This attribution is complicated by the extremely high prevalence of these infections in the community and the usual vague history of early childhood disease.

Tuberculosis has an important role in the development of bronchiectasis and may occur as a consequence of widespread parenchymal damage. Whitwell showed that the chronic peribronchial lymphadenopathy that occurs with this condition may cause localised bronchial obstruction (particularly in the right middle and upper lobes) leading to secondary bacterial infection [7]. Tuberculosis is a leading cause of bronchiectasis in Eastern Europe and Asia [22].

Viral infection may also have a potential role in the development of bronchiectasis. Human immunodeficiency virus (HIV) is associated with an increased risk of bronchiectasis particularly in the developing world with poor access to antiretroviral therapy [23]. In Indigenous Australians human T-lymphotropic virus 1 (HTLV-1) infection is associated with bronchiectasis [24]. Becroft has described that adenovirus infection in childhood may be followed by the development of bronchiectasis [25]. Acute viral infection may also potentially have a triggering role and this will be discussed in more detail below.

### 4.2. Immune Deficiency

Hypogammaglobulinaemia has an important role in the development of bronchiectasis. This has most commonly been described in the context of common variable immune deficiency (CVID) with low levels of immunoglobulin (Ig) G and less commonly in X-linked agammaglobulinaemia. Two studies have demonstrated a high prevalence of bronchiectasis in this group of patients [26, 27]. There may also be an association with IgG subclass deficiency but this is controversial [20, 21, 28, 29]. Deficiency of IgA both systemic and secretory may have a role but as most patients with IgA deficiency have no clinical disease this association remains to be proven. Further functional studies of antibody production to vaccines may give useful information about the clinical relevance of immunoglobulin deficiency [20, 29].

Deficiency of the transporter associated with antigen processing (TAP) affects the function of the major histocompatibility complex class I (MHC-I). Without a functional TAP, most human leukocyte antigen (HLA) class I molecules are not expressed on the cell surface. Such patients have severe upper and lower respiratory tract infections and may develop bronchiectasis [30].

Mannose binding lectin (MBL) is expressed in the blood as an acute phase reactant and binds to mannose on the surface of bacterial pathogens. It can then activate complement by the lectin pathway. Its deficiency has been described to be associated with bronchiectasis [31]. The functional significance of its deficiency remains to be determined.

Hyper-IgE syndrome is a primary immunodeficiency with eczema, recurrent skin and lung infections, skeletal/connective tissue abnormalities, and raised IgE. An important pulmonary manifestation is bronchiectasis [32]. One cause of this syndrome is mutation of signal transducer and activator of transcription 3 (STAT3). This STAT3 affects the production of a variety of cytokines such as impairment of interleukin (IL) 17 production.

Malignancy has wide ranging effects on the function of the immune system. Bronchiectasis has been described to occur in children who are in remission from acute lymphoblastic leukaemia on maintenance chemotherapy [33]. Chronic lymphatic leukaemia may be associated with hypogammaglobulinaemia and bronchiectasis [34]. Another recent study has described the development of bronchiectasis in hematologic malignancy [35].

### 4.3. Mucociliary Function

The mucociliary apparatus has a key role in the innate immune response in the lung.

Ciliary dyskinesia occurs when there is a deficiency in a dynein fragment in the cilia which are no longer able to beat normally. This affects both the upper and lower respiratory tracts and most patients will have severe sinus disease [36, 37]. In addition as the cilia are present in the reproductive tract, infertility occurs in patients with mucociliary disorders. Kartagener's syndrome is a form of ciliary dyskinesia in which there is situs inversus (e.g., heart is on the right side of the body rather than the left) [37]. There is another less-defined entity with defective mucus production “Young's syndrome” that has been described previously although its role in bronchiectasis has not clearly been established [38].

### 4.4. Systemic Inflammatory Disease

There is a high prevalence of bronchiectasis in patients with rheumatoid arthritis (RA). Up to 30% of patients with RA will have changes of bronchiectasis on HRCT [39, 40] and the incidence of symptomatic airway infection may be of the order of 10% [41]. Bronchiectasis may be manifest before the onset of joint disease. Demoruelle et al. studied patients who had increased levels of RA autoantibody expression without clinical disease and found airway changes in 76% and bronchiectasis in 14% [42]. Patients with bronchiectasis have a high prevalence of RA autoantibody expression [43]. There may be shared mucosal inflammatory pathways between the lung and the joints. Periodontal bacterial infection may have a triggering role in RA. The immunosuppression required to treat this condition may also be a potential risk factor for the development of respiratory infection. Other arthritic diseases including systemic lupus erythematosus [44], scleroderma [45], and Sjogren's syndrome [46] are associated with bronchiectasis.

Inflammatory bowel disease including Crohn's disease and ulcerative colitis is associated with an increased risk of bronchiectasis [47]. As discussed above, the interaction of the GIT and lung immune responses is a topic of interest. In relation to this, colectomy for ulcerative colitis has been described to lead to the development of severe bronchiectasis [48].

### 4.5. Airway Obstruction

Airway obstruction may lead to persistent infection and the development of localised bronchiectasis. This has been best described in the context of an inhaled foreign body as may occur in children [49]. Airway obstruction from a primary lung tumor in the airway lumen may also have a similar effect [50, 51]. These findings demonstrate the importance of obstruction and retained secretions more broadly in the context of bronchiectasis as emphasised by Cole.

### 4.6. Gastroesophageal Reflux/Aspiration

The microaspiration of PPMs from the upper airway is a primary route leading to lower respiratory tract infection. The role of gastroesophageal reflux in the pathogenesis of bronchiectasis has been unclear. Aspiration of chemicals such as ammonia can cause airway stricturing and bronchiectasis [52, 53]. Bronchiectasis following heroin overdose may arise from aspiration or septic emboli [54, 55].

### 4.7. Chronic Obstructive Pulmonary Disease

It has been recently recognised that bronchiectasis is a common complication of COPD. There have been a number of published studies which have described the prevalence of COPD in bronchiectasis to range from 29% to 67% [4, 6, 56]. Therefore based on these studies it may be that bronchiectasis affects many of millions of people (worldwide, approximately 175 million people have COPD [57]). In addition, patients with COPD who have concurrent bronchiectasis have worse disease, more frequent exacerbations/hospitalisations, and higher mortality.

### 4.8. Other Factors

Alpha-1-antitrypsin deficiency leads to damage to the lung by excess activity of the protease trypsin. It is most commonly associated with the development of emphysema, which in approximately 30% of patients is associated with localised bronchiectasis [58].

There have been a number of rare conditions that have been described to be associated with the development of bronchiectasis; some of these include Pink's disease (lead poisoning), yellow nail syndrome, congenital abnormalities of airway cartilage, and toxic gas inhalation. Two important factors that are relevant to the development of bronchiectasis are age and economic/social disadvantage.

There is a higher incidence of infection that occurs in younger children before the immune system matures and also in older adults who have a less effective immune response. This may also be relevant to the inflammatory process in bronchiectasis. Field followed up a large cohort of children with childhood bronchiectasis and found that children had less severe clinical symptoms upon reaching adolescence/adulthood [59–61]. Another study of adults found that children whose symptoms has improved tended to have a recurrence of their symptoms over the age of 50 years. There was also a second cohort of previously healthy adults who first developed symptoms of bronchiectasis over the age of 50 years [62].

Social/economic disadvantage has been shown to be closely correlated with the development of bronchiectasis in indigenous populations including Australian aborigines, New Zealand Maoris/Pacific islanders, and Alaskan Eskimos [63–66]. Multiple factors have been proposed to explain this increased incidence.

## 5. Inflammatory Response

Bronchiectasis is characterised by a persistent inflammatory response to airway infection. This inflammatory response is typically directed against opportunistic bacteria in the lung. These same microorganisms appear to exist as commensals in the nasopharynx. Why these bacteria generate a different immune response in these adjacent locations is not understood. This section will review both innate and adaptive immune responses and the response to some specific pathogens.

### 5.1. Innate Immunity

#### 5.1.1. Neutrophils

The published literature has concentrated on the neutrophil as a driver of innate immune responses in bronchiectasis. Neutrophils are found in large numbers in both stable and exacerbated bronchiectasis [16, 67]. Neutrophils use surface receptors to recognise bacterial structures and pathogen-associated molecular patterns (PAMPs). The toll-like receptors (TLRs) are the most well-defined receptors to bacterial infection, especially TLR2 and TLR4. The dominant bacterial pathogens in bronchiectasis have all been shown to activate TLRs [68]. TLR4 deficient mice have impaired clearance of* H. influenzae *[69]. There is a lack of specific studies in bronchiectasis although the one report described increased expression of TLR2 suggesting there may be differential TLR effects [70].

Activated neutrophils phagocytose bacteria. The neutrophils then use a variety of methods to kill intracellular bacteria. Arguably the most important phagocyte microbicidal mechanism is the respiratory oxidative burst which creates reactive oxygen species (ROS) such as hydrogen peroxide. The intracellular ROS is highly effective in mediating killing and its deficiency as occurs in the inherited condition chronic granulomatous disease (CGD) results in repeated severe infections. Whether there is impairment in ROS production in bronchiectasis remains controversial with both reduced [21] and normal [20] responses being reported. Bronchiectasis has been reported to occur in patients with CGD [71]. The ROS are very permeable and may leak out of the neutrophil and damage the adjacent lung tissue.

Activation of innate immune responses in bronchiectasis causes the releases of chemokines which significantly increases the cellular inflammatory infiltrate including IL-6, IL-8, and leukotriene B4 [72–74]. There is also increased production of inflammatory cytokines tumor necrosis factor alpha (TNF-*α*) and IL-1*β* and adhesion molecules such as E-selectin in the airways of patients with bronchiectasis [75, 76].

#### 5.1.2. Macrophages

Macrophages have similar function to neutrophils with their expression of surface receptors such as TLRs, phagocytosis, and intracellular killing including the production of ROS. They are the dominant cell in the steady state and may have a more important role in the chronic inflammatory state as opposed to acute exacerbations in which neutrophils may be more important. There is a relative lack of published data about the role of macrophages in bronchiectasis. Zheng et al. have shown increased macrophage numbers in endobronchial biopsies in patients with bronchiectasis when compared to control [75]. They proposed that these lung macrophages may induce an infiltration of neutrophils via TNF-*α* production. Studies have described that phagocytosis of bacteria is impaired in patients with COPD [77, 78]. A recent study has reported that phagocytosis by alveolar macrophages is also reduced in bronchiectasis [32].

#### 5.1.3. Protease Imbalance

**Figure 2:**
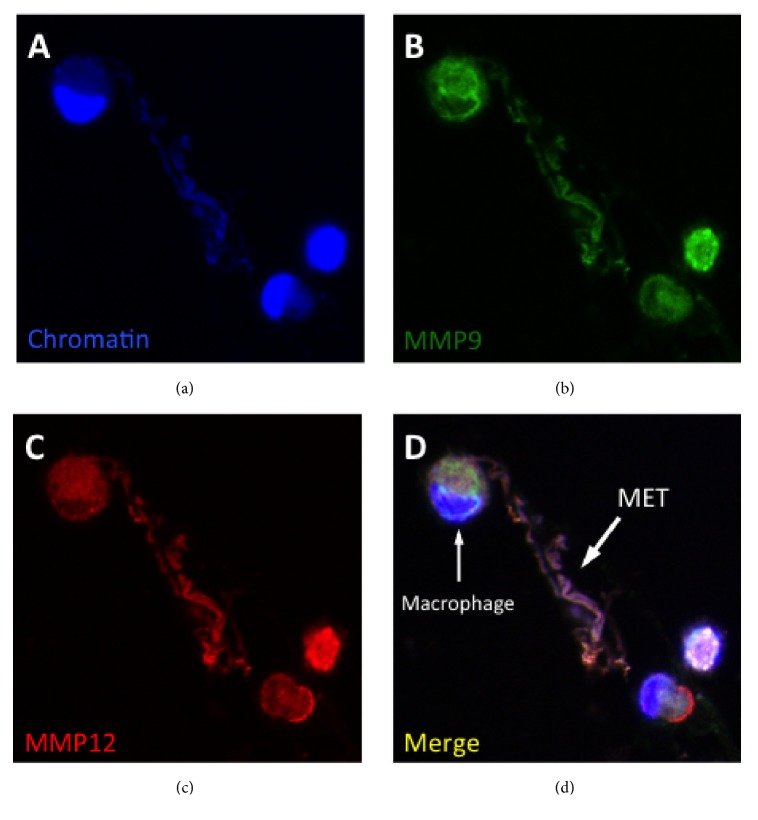
*Protease expression by phagocyte extracellular trap.* The figure shows a human alveolar macrophage expressing an extracellular traps (MET). (a) shows expression of extracellular chromatin, (b) matrix metalloproteinase (MMP) 9, (c) MMP12, and (d) the merged image.

Protease imbalance is characterised by excessive production of proteases and/or deficiency of inhibitors such as *α*-1 antitrypsin. Protease imbalance has a key role in the pathogenesis of COPD and bronchiectasis. Proteases are principally produced by the lung phagocytes and include neutrophil elastase (NE) and macrophage matrix metalloproteinases (MMP) 1, 9, and 12. They are probably the main mediators that damage the bronchial wall and leads to the pathological bronchial dilation that is the cardinal feature of bronchiectasis [79, 80]. Proteases are proinflammatory and correlate with sputum volume, lung function, and extent of radiologic disease [81]. Bacterial pathogens may also secrete proteases [82, 83]. How these proteases are expressed in bronchiectasis is not well understood but one potentially relevant mechanism is via the expression of phagocyte extracellular traps (Figure 2). Neutrophil extracellular traps (NETs) are induced in response to bacterial infection and other stimuli and are comprised of extracellular processed chromatin with granular proteases such as NE [84]. These NETs have an important bactericidal function but also may be potentially damaging to the lung parenchyma. In addition, macrophage extracellular traps (METs) have been recently described. These may be produced in the lung in response to relevant stimuli such as* H. influenzae* and cigarette smoke [85, 86].

#### 5.1.4. Other Immune Cells

There may be association between eosinophils and bronchiectasis [87]. In a cohort of patients with idiopathic bronchiectasis, Boyton et al. showed that there was HLA-C group 1 homozygosity [88]. Analysis of the relationship between HLA-C and killer cell immunoglobulin-like receptors (KIR0 genes) suggested a shift to activatory NK cell function. NK cells serve as a bridge between the innate and adaptive immune responses and may also contribute to the bronchial lymphocyte infiltration described below. A subsequent study demonstrated a lack of association between KIR and HLA-C type and susceptibility to idiopathic bronchiectasis [89]. The diverging conclusions in these two studies could potentially be due to the use of different control groups.

### 5.2. Adaptive Immunity

In his seminal study, Whitwell showed that the small airways of patients with bronchiectasis have a prominent lymphocyte infiltrate with the formation of lymphoid follicles [7]. Other studies have also demonstrated T cell infiltration in bronchiectasis [90, 91]. A recent study of human surgical lung specimens in patients with bronchiectasis found numerous peribronchial lymphoid aggregates containing B-lymphocytes, T-lymphocytes, and germinal centres [92].

Th17 immune responses activate neutrophils and have an important role in host defence to bacteria. They also contribute to inflammation and have been proposed to have an important role in the pathogenesis of bronchiectasis [93]. Increased levels of IL-17 and Th17 cells in the bronchial epithelium and in endobronchial biopsies have been described [92, 94].

Nontypeable* Haemophilus influenzae* (NTHi) is the most common bacterium found in patients with bronchiectasis. This bacterium is highly adapted to the lung and may under certain circumstances be able to live intracellularly [95]. Both healthy control subjects and patients with chronic NTHi infection make specific antibody which is effective in mediating bacterial extracellular killing [96]. Normal adult control subjects make a Th1 predominant response to this bacterium whilst subjects with bronchiectasis and persistent infection with NTHi have a Th2 response [97]. Similar findings have been noted in a pediatric population [98]. In addition patients with COPD have been found to have similar findings [99]. A Th1 response is the classical immune response involved in the clearance of intracellular infection. A deficient Th1 (or instead a Th2 response) has been described to occur in nonclearing immunity to* Leishmania* [100] and mycobacterial infection [101]. A Th1 immune response is generally more inflammatory than a Th2 response and Wynn has proposed that in chronic inflammatory disease the downregulation of Th1 immunity may potentially reduce host damage [102].

Infection with* Pseudomonas aeruginosa* is a major feature of more advanced bronchiectasis. Quigley et al. studied a cohort of patients and assessed immune responses to a* P. aeruginosa* antigen [103]. They found a relative reduction in Th1 polarizing transcription factors but enhanced immunity with respect to antibody production, innate cytokines, and chemokines.

Infection with the fungal species* Aspergillus *may cause disease in susceptible individuals. This fungus is a very prevalent environmental organism that is frequently inhaled and may colonise the airways but it appears to be generally a commensal. However this fungus causes allergic bronchopulmonary aspergillosis (ABPA). This is characterised by a strong immune response to* Aspergillus* spp. as assessed by skin prick reactivity or the presence of specific antibodies and high levels of IgE. Such patients have asthma and frequently bronchiectasis [104]. The mechanisms of ABPA are not well understood but such patients appear to have a hypersensitive Th2 response to this prevalent environmental fungus.

## 6. Triggering Factors

As discussed above, Cole proposed that a triggering factor was important in the initiation of bronchiectasis. This triggering factor has not been clearly defined in the published literature. It does imply that there is a discrete event that occurs and from this time onwards the airways remain colonised by bacteria with associated inflammation. Two factors may be relevant to this: acute severe chest infections such as pneumonia and viral infection.

The most commonly identified cause for bronchiectasis is postinfective. Certain infections such as early childhood pertussis infection or tuberculosis may cause significant structural lung damage. Most infections such as pneumonia do not cause obvious structural lung damage; however in a proportion they may be followed by persistent bronchitis which could lead to the development of bronchiectasis (particularly in a patient with underlying immune deficiency).

Viral infections could also have a role in triggering exacerbations and potentially in the initiation of airway infection. Airway bacterial infection may occur as a complication of influenza and in the 1918 pandemic may have been the leading cause of death [105]. Infection of a cohort of COPD patients with rhinovirus was associated with a high incidence of secondary bacterial infection [106]. There are very few studies that have assessed the role of viral infection in bronchiectasis. Two recent studies have described that approximately half of exacerbations of bronchiectasis were associated with viral infection [107, 108]. Another study detected viral infection in 44% of clinically stable children with bronchiectasis [109].

## 7. Assessment and Investigation of the Patient with Bronchiectasis

Well-defined guidelines are available for the assessment of patients with bronchiectasis [110–112]. Details should be obtained about the frequency, color, and amount of sputum production, associated respiratory symptoms (dyspnoea, chest pain, and hemoptysis), exacerbations, and upper airway symptoms (a feature of childhood onset disease). The most common finding on examination is the presence of chest crackles.

Tests of immune function should concentrate on those which are likely to change patient management. All subjects should have a full blood examination, immunoglobulin levels (especially IgG and IgE), and specific tests for the presence of* Aspergillus* (e.g., precipitins or specific IgG). Consideration could also be given to more detailed testing of humoral immunity (vaccine responses, IgG subclasses, etc.) and measurement of a-1 antitrypsin levels. In high-risk populations testing for HIV or HTLV-1 infection may be appropriate. It is also important to obtain good quality of lower respiratory tract specimens for microbial analysis.

## 8. Management of the Patient with Bronchiectasis

Key areas in the management of patients with bronchiectasis include appropriate use of antibiotics, sputum clearance, vaccination, and optimising patient fitness and nutrition; these have been listed in a variety of national guidelines [110–112]. This section will concentrate on those areas which are directly relevant to immunology. Any agent which may alter and particularly suppress an immune response can theoretically make infection worse and this is an important consideration in the use of immunomodulatory therapies for the treatment of bronchiectasis.

In patients who have low levels of IgG, replacement therapy has been shown to reduce the frequency of infections and slow progression of disease [113, 114]. Despite replacement therapy some patients may have progressive disease [115]. This is typically given as monthly infusions of IgG. Patients need to be monitored for the development of allergic reactions in the immediate transfusion period. Consideration can also be given to the administration of replacement IgG in patients with defective antibody to stimuli such as vaccines and potentially in patients with IgG subclass deficiency.

Patients with ABPA are usually treated with systemic corticosteroids and also potentially with an antifungal agent such as itraconazole [104]. In addition to infection with* Aspergillus* spp. patients with ABPA may develop infection with other microbial pathogens such as* P. aeruginosa* and this complicates the use of corticosteroids.

As bronchiectasis is characterised by lung inflammation, the use of anti-inflammatory agents is theoretically likely to be useful. However systematic reviews of the use of effect of nonsteroidal anti-inflammatory agents and corticosteroids have failed to show clear benefit in the treatment of bronchiectasis [111, 116].

Macrolides have been shown to have anti-inflammatory effects in addition to their antibiotic actions. Three high-quality clinical trials have demonstrated improved outcomes with the use of macrolides and, with decreased exacerbations, improved symptoms and lung function [117–119]. However there are concerns about the overuse of these antibiotics and the development of bacterial resistance. Nonantibiotic macrolides have been developed and are currently in clinical trials. A recent report describes that nonantibiotic macrolides restore phagocytic function in vitro in alveolar macrophages [120].

Inhibition of proteases has been of considerable interest as this is a primary mediator involved in the pathogenesis of bronchiectasis. Research has concentrated on neutrophil elastase and this topic has recently been reviewed by Polverino et al. [121]. Generally the trials have not shown any conclusive effects on improving outcome in a variety of inflammatory lung conditions. Nebulised deoxyribonuclease (DNase) 1 in the form of dornase alfa (or Pulmozye™) breaks down bacterial DNA and has been used to improve sputum clearance in patients with CF [122]. A randomised trial in patients with bronchiectasis found that the use of dornase alfa was associated with worse outcomes [123]. Bacterial infections induce the formation of phagocyte extracellular traps which express pathogenic mediators such as proteases and their expression is dismantled by the addition of DNase 1 [86, 124]. Therefore the use of DNase may have a potential role as an agent to inhibit pathogenic protease expression perhaps in combination with an antibiotic.

Recent guidelines have highlighted the importance of vaccination in the management of patients with bronchiectasis, mainly the use of influenza and pneumococcal vaccines. Further understanding of the immune response in bronchiectasis will be important in the development of vaccines. A way forward in this regard would be to further define protective immunity to key bacterial pathogens. The pneumococcal polysaccharide vaccine has been available for many years but a new conjugate vaccine may be more effective [125]; the role of this conjugate vaccine in bronchiectasis remains to be defined. As the vast majority of* H. influenzae* infections are with nontypeable strains, the HiB vaccine is not generally used in patients with bronchiectasis. There is no standard vaccine used against NTHi. Pizzuto et al. have shown that vaccination with a PCV with a single NTHi vaccine was associated with higher Th-1 responses, which is theoretically protective against this bacterium [126]. There are minimal relevant studies in the literature about the use of vaccinations to treat infections with* M. catarrhalis* or* P. aeruginosa, *although potential experimental vaccine candidates are available.

## 9. Conclusions

Bronchiectasis is a very prevalent condition that is a major cause of respiratory morbidity. It is heterogeneous and has a wide variety of potential causes, all of which are associated with impairment in the host response to infection. This may lead to the establishment of chronic airway infection and consequent inflammation. Patients will generally have persistent disease despite aggressive use of antibiotics and optimal sputum clearance methods. New therapies based on manipulating the immune response are becoming available and offer significant promise for the management of this condition.

## Figures and Tables

**Table 1:** Important predisposing causes for bronchiectasis.

Postinfective
Childhood infection: pneumonia, measles, whooping cough
Tuberculosis
Viral infection: HIV, HTLV-1
Immune deficiency
Humoral immunity: CVID, X-linked agammaglobulinaemia
?IgG subclass
Transporter associated with antigen processing deficiency
Mannose binding lectin deficiency
Hyper-IgE syndrome
Chronic granulomatous disease
Malignancy
Mucociliary function
Ciliary dyskinesia
Systemic inflammatory disease
Rheumatoid arthritis
Other arthritic disorders
Inflammatory bowel disease
Airway obstruction
Foreign body
Airway tumor
Gastroesophageal reflux/aspiration
Chronic obstructive pulmonary disease
Other factors
Alpha-1-antitrypsin deficiency
Extremes of age
Economic/social disadvantage

HIV: human immunodeficiency virus. HTLV-1: human T-lymphotropic virus 1. CVID: common variable immune deficiency.
